# Supplementary material for: Causal Relationship between Aging and Anorexia Nervosa: A White-Matter-Microstructure-Mediated Mendelian Randomization Analysis
Source: Biomedicines. 2024 Aug 16;12(8):1874. doi: 10.3390/biomedicines12081874 (PMC11351342; doi:10.3390/biomedicines12081874)
Supplement: Supplementary file 1 [file biomedicines-12-01874-s001.zip › Supplementary Figure/Figure S4/Singe SNP plot of MR analysis for TL to ubm-b-1624.pdf]

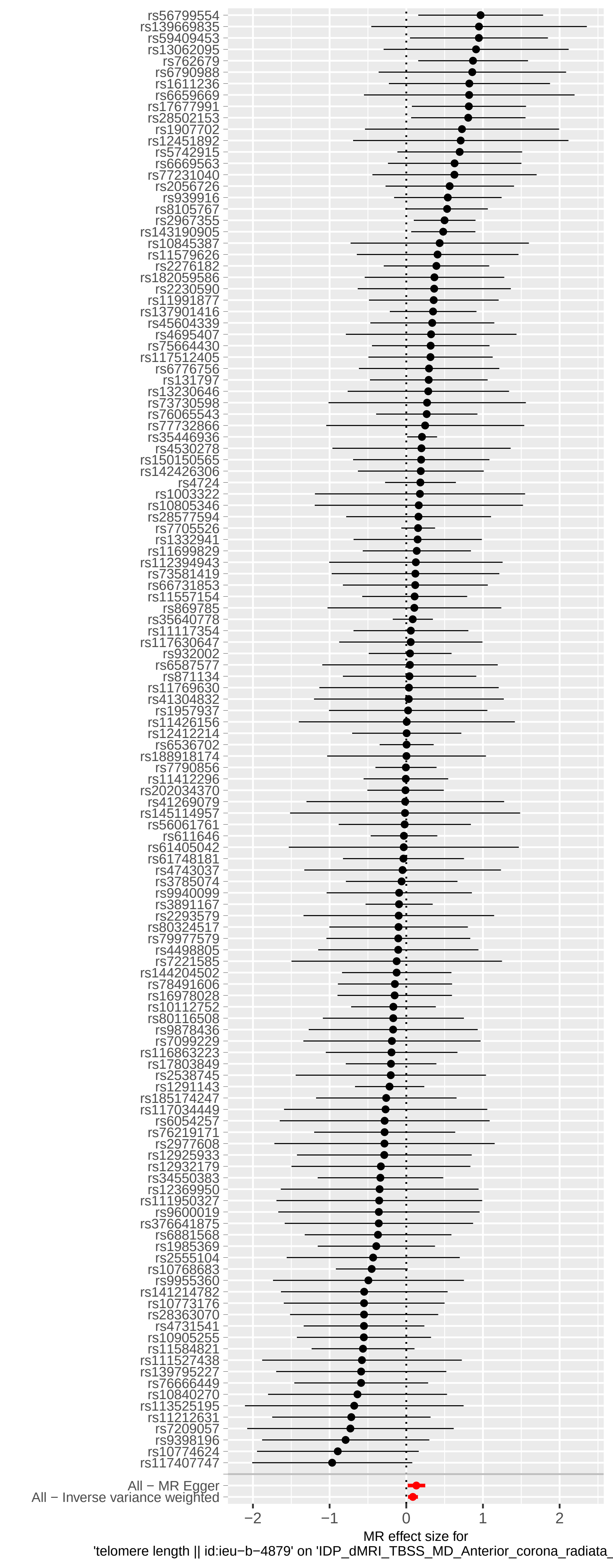

rs56799554
rs139669835
rs59409453
rs13062095
rs762679
rs6790988
rs1611236
rs6659669
rs17677991
rs28502153
rs1907702
rs12451892
rs5742915
rs6669563
rs77231040
rs2056726
rs939916
rs8105767
rs2967355
rs143190905
rs10845387
rs11579626
rs2276182
rs182059586
rs2230590
rs11991877
rs137901416
rs45604339
rs4695407
rs75664430
rs117512405
rs6776756
rs131797
rs13230646
rs73730598
rs76065543
rs77732866
rs35446936
rs4530278
rs150150565
rs142426306
rs4724
rs1003322
rs10805346
rs28577594
rs7705526
rs1332941
rs11699829
rs112394943
rs73581419
rs66731853
rs11557154
rs869785
rs35640778
rs11117354
rs117630647
rs932002
rs6587577
rs871134
rs11769630
rs41304832
rs1957937
rs11426156
rs12412214
rs6536702
rs188918174
rs7790856
rs11412296
rs202034370
rs41269079
rs145114957
rs56061761
rs611646
rs61405042
rs61748181
rs4743037
rs3785074
rs9940099
rs3891167
rs2293579
rs80324517
rs79977579
rs4498805
rs7221585
rs144204502
rs78491606
rs16978028
rs10112752
rs80116508
rs9878436
rs7099229
rs116863223
rs17803849
rs2538745
rs1291143
rs185174247
rs117034449
rs6054257
rs76219171
rs2977608
rs12925933
rs12932179
rs34550383
rs12369950
rs111950327
rs9600019
rs376641875
rs6881568
rs1985369
rs2555104
rs10768683
rs9955360
rs141214782
rs10773176
rs28363070
rs4731541
rs10905255
rs11584821
rs111527438
rs139795227
rs76666449
rs10840270
rs113525195
rs11212631
rs7209057
rs9398196
rs10774624
rs117407747
All − MR Egger
All − Inverse variance weighted
−2
−1
0
1
2
MR effect size for
'telomere length || id:ieu−b−4879' on 'IDP_dMRI_TBSS_MD_Anterior_corona_radiata_
